# The Ca^2+^-activated cation channel TRPM4 is a negative regulator of angiotensin II-induced cardiac hypertrophy

**DOI:** 10.1007/s00395-015-0501-x

**Published:** 2015-06-05

**Authors:** Miklós Kecskés, Griet Jacobs, Sara Kerselaers, Ninda Syam, Aurélie Menigoz, Peter Vangheluwe, Marc Freichel, Veit Flockerzi, Thomas Voets, Rudi Vennekens

**Affiliations:** Laboratory of Ion Channel Research, Department of Molecular and Cellular Medicine, KU Leuven, Campus Gasthuisberg, Herestraat 49, 3000 Leuven, Belgium; TRP Research Platform Leuven (TRPLe), KU Leuven, Campus Gasthuisberg, Herestraat 49, 3000 Leuven, Belgium; Laboratory of Cellular Transport Systems, Department of Molecular and Cellular Medicine, KU Leuven, Campus Gasthuisberg, Herestraat 49, 3000 Leuven, Belgium; Pharmakologisches Institut, Universität Heidelberg, Im Neuenheimer Feld 366, 69120 Heidelberg, Germany; Experimentelle und Klinische Pharmakologie und Toxikologie, Universität des Saarlandes, Geb. 46, 66421 Homburg, Germany

**Keywords:** Hypertrophy, TRPM4, Angiotensin, Calcineurin, Store-operated calcium entry

## Abstract

Cardiac muscle adapts to hemodynamic stress by altering myocyte size and function, resulting in cardiac hypertrophy. Alteration in myocyte calcium homeostasis is known to be an initial signal in cardiac hypertrophy signaling. Transient receptor potential melastatin 4 protein (TRPM4) is a calcium-activated non-selective cation channel, which plays a role in regulating calcium influx and calcium-dependent cell functions in many cell types including cardiomyocytes. Selective deletion of TRPM4 from the heart muscle in mice resulted in an increased hypertrophic growth after chronic angiotensin (AngII) treatment, compared to WT mice. The enhanced hypertrophic response was also traceable by the increased expression of hypertrophy-related genes like Rcan1, ANP, and α-Actin. Intracellular calcium measurements on isolated ventricular myocytes showed significantly increased store-operated calcium entry upon AngII treatment in myocytes lacking the TRPM4 channel. Elevated intracellular calcium is a key factor in the development of pathological cardiac hypertrophy, leading to the activation of intracellular signaling pathways. In agreement with this, we observed significantly higher Rcan1 mRNA level, calcineurin enzyme activity and protein level in lysates from TRPM4-deficient mice heart compared to WT after AngII treatment. Collectively, these observations are consistent with a model in which TRPM4 is a regulator of calcium homeostasis in cardiomyocytes after AngII stimulation. TRPM4 contributes to the regulation of driving force for store-operated calcium entry and thereby the activation of the calcineurin–NFAT pathway and the development of pathological hypertrophy.

**Electronic supplementary material:**

The online version of this article (doi:10.1007/s00395-015-0501-x) contains supplementary material, which is available to authorized users.

## Introduction

Cardiac hypertrophy is characterized by an increase in heart mass and associated changes in the shape of the left ventricle [13]. The defining signs of pathological cardiac hypertrophy are an increase in cardiomyocyte size, enhanced protein synthesis, and an increase in cardiac fibroblast proliferation [9]. Pathological hypertrophy can be triggered by humoral stimuli such as angiotensin II, or conditions such as hypertension, valvular dysfunction and myocardial infarction [8]. In general, hypertrophic growth is considered as an adaptive response to pathological stimuli, to preserve pump function of the heart. However, prolonged hypertrophy is associated with a significant increase in the risk for sudden death or progression to heart failure. In the past decades, many intracellular signaling pathways have been shown to be involved in hypertrophic response including MAPK, calcineurin–NFAT, CaMKII–HDAC, PKC alpha, ERKs and JNKs [14]. Although signal-transduction pathways are inherently complex and abundant, one of the most common initial events in the onset of hypertrophy is an alteration in Ca^2+^ homeostasis. Elevated systolic and/or diastolic [Ca^2+^]_*i*_ can activate intracellular cascades leading to hypertrophic growth. Obviously, cardiomyocytes exhibit dramatic fluctuations of [Ca^2+^]_*i*_ during the cardiac cycle, making it difficult to explain how Ca^2+^-activated signaling proteins function. One possibility is the presence of specialized pools of Ca^2+^ which are spatially separated from the bulk Ca^2+^. However, the source of this subcellular Ca^2+^ still remains doubtful. Growing evidence points to voltage-gated Ca^2+^ channels, i.e., L-type, T-type [3, 23], as well as TRPC channels [10, 33] as the source of this Ca^2+^. In addition, recent studies also suggested store-operated calcium entry (SOCE) as an important mechanism in cardiac hypertrophy signaling [15, 16, 32].

The transient receptor potential (TRP) superfamily of ion channels forms a large and diverse family of cation channels related to the product of the *trp* gene in *Drosophila.* In the heart, the presence of several TRPs has been reported, including TRPC1, TRPC3-7, TRPV1, TRPV2, TRPV4, TRPM4, TRPM6 and TRPP2 [19]. TRPM4, a member of the TRPM subfamily, is a calcium-activated non-selective cation channel. The channel is equally permeable to Na^+^ and K^+^, but impermeable to Ca^2+^. The calcium sensitivity can be modulated by protein kinase C (PKC)-mediated phosphorylation, cellular PIP_2_ levels and by membrane depolarization [31]. In humans and also in rodents, TRPM4 has been detected both in atria and ventricles [39], and also in vascular endothelium and smooth muscle [7]. Recently, we have shown that TRPM4 channel activity can regulate cardiac contractility in the mouse heart. The lack of the channel results in shortening of action potential duration and an increased Ca^2+^ current via voltage-gated Ca^2+^ channels, which becomes even more prominent during beta-adrenergic stimulation in ventricular myocytes [24, 36], and which is especially relevant during heart failure [20]. A role for TRPM4 has been suggested in several physiological and pathological processes in the cardiovascular system [22]. TRPM4 is overexpressed in the hypertrophied hearts of spontaneously hypertensive rats, but the physiological function of TRPM4 in this process is unclear [5]. Recently, mutations in the TRPM4 gene, which are associated with Brugada syndrome, long QT syndrome and cardiac conduction defects, such as Progressive familiar heart block type I [21, 26, 34], have been reported. Also, it was shown that TRPM4-deficient mice display moderate hypertension, which is a result of the increased level of catecholamines in blood plasma of TRPM4-deficient mice compared to WT [25].

In the current study, we show that TRPM4 fine-tunes the amount of Ca^2+^ entry via store-operated Ca^2+^ channels into cardiomyocytes after chronic AngII stimulation, thereby determining the degree of calcineurin–NFAT activation, which is a signaling cascade that is both sufficient and required for the hypertrophic response [28].

## Results

### Generation of cardiac-specific TRPM4 knockout mouse

**Fig. 1:**
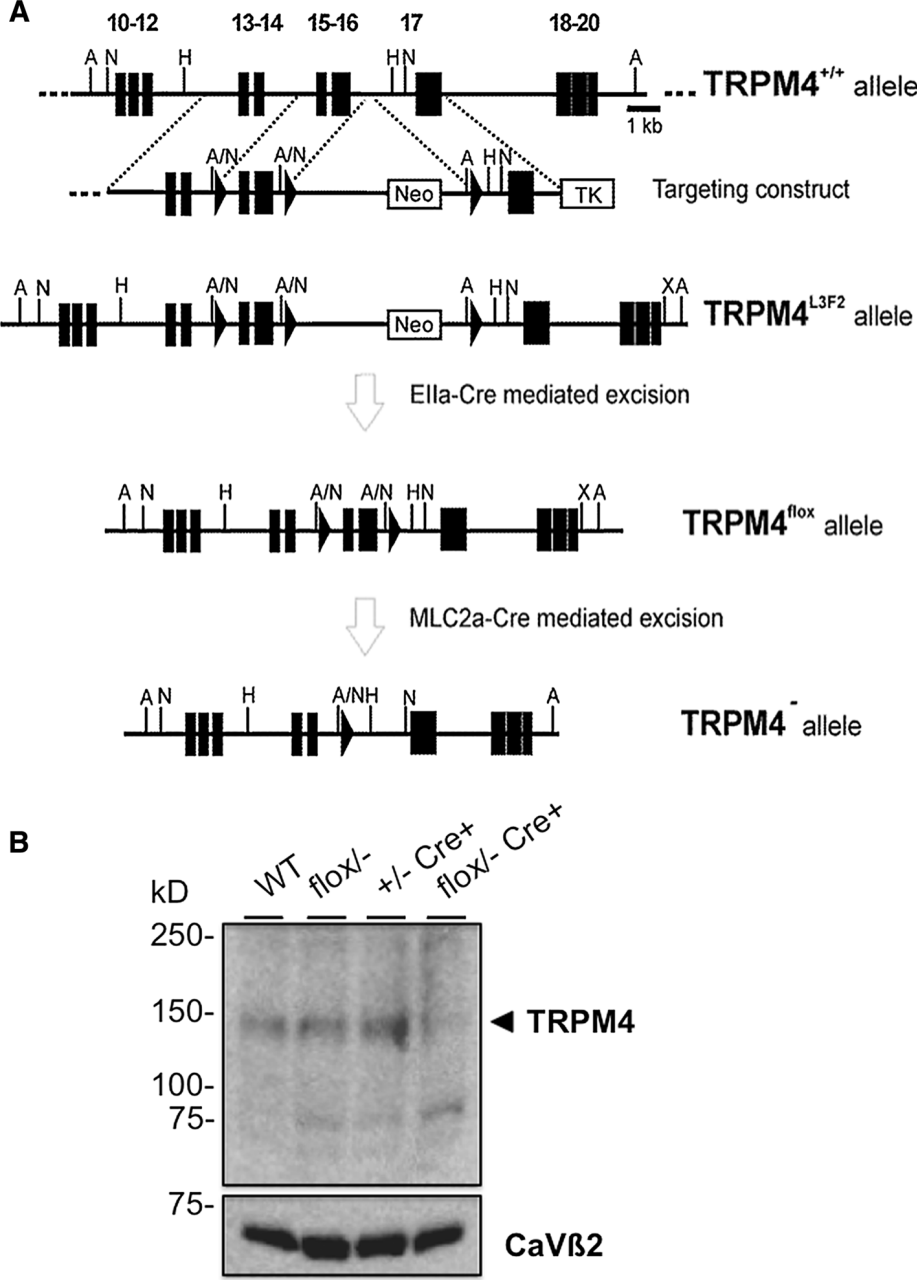
Generation of cardiac-specific TRPM4 knockout mice (*Trpm4* ^*cKO*^). **a** Targeting of the TRPM4 gene, as described previously [40]. To obtain the cardiac-specific knockout, *Trpm4* ^flox/flox^ animals were crossed with MLC2a-Cre mice. See the text for more details. **b** Representative Western blot of protein fractions from the left ventricle of WT, flox/−, +/−Cre+ and cardiac-specific TRPM4-deficient (flox/− Cre+) mice using TRPM4-specific antibody

Considering that hypertension and increased plasma catecholamine levels, which can influence the tendency to develop hypertrophy, were recently reported in global TRPM4-deficient mice [25], we have generated a cardiac-specific TRPM4 knockout mouse line (*Trpm4*^cKO^), through crossbreeding of *Trpm4*^flox^ mice with MLC2a-Cre mice [40] (Fig. 1a). This results in cardiac-specific deletion of exon 15 and 16 from the *Trpm4* gene, which encode for the first transmembrane domain of the TRPM4 protein [37]. Western blotting confirmed deletion of the TRPM4 protein from the heart of *Trpm4*^cKO^ mice (Fig. 1b). To compare blood pressure in awake, freely moving WT and *Trpm4*^*cKO*^ mice, we implanted telemetric devices. Blood pressure and heart rate as measured over the course of 2 weeks after transmitter implantation were essentially identical in *Trpm4*^cKO^ mice and MLC2a-Cre-negative *Trpm4*^flox/−^ mice (Supplementary Figure 1).

### TRPM4 deficiency in myocytes causes increased cardiac hypertrophy after chronic angiotensin treatment

**Fig. 2:**
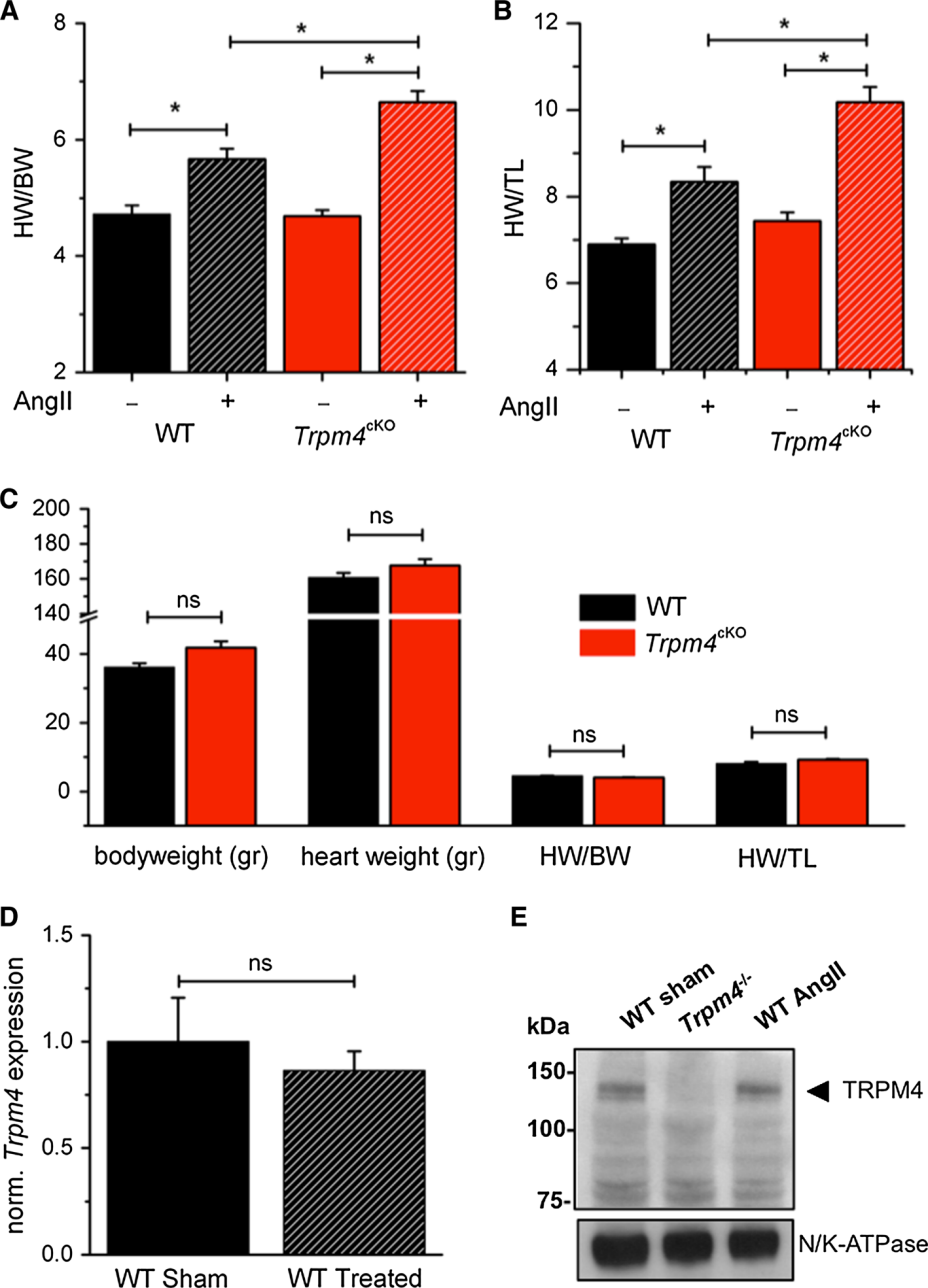
*Trpm4* ^*cKO*^ mice display increased heart size compared to WT upon AngII treatment. **a** Heart weight/body weight (HW/BW) and **b** heart weight/tibial length (HW/TL) ratios of WT and *Trpm4* ^*cKO*^ mice after sham or Ang II (3 mg/kg/day during 2 weeks via osmotic pumps) treatment. At the time of the experiment (8–12 weeks of age), body weight was not different between groups. (mean ± SEM; WT sham: *n* = 7; WT treated *n* = 10; *Trpm4* ^cKO^ sham: *n* = 7; *Trpm4* ^cKO^ *n* = 10; *p < 0.05). **c** Baseline hypertrophy as developed with aging (1 year old) (mean ± SEM; *n* = 5 for each group). **d** TRPM4-specific qPCR and Western blot (**e**) show no difference in TRPM4 mRNA and protein expression level after AngII treatment in WT mice (*n* = 4). A cardiac muscle lysate from *Trpm4* ^−/−^ mice was added to underline the specificity of the antibody (*right panel*, *middle* *lane*)

In previous studies, we have shown by different approaches that TRPM4 protein is expressed in mouse heart, both in ventricles and atria, but absent in TRPM4 knockout mice [25]. Western blot analysis showed clear expression in hearts of WT and Cre^−^ Trpm4^flox/−^ mice, but TRPM4 was absent in the heart of Cre^+^*Trpm4*^flox/−^ mice (*Trpm4*^*cKO*^, Fig. 1b). To evaluate the effect of TRPM4 on the development of cardiac hypertrophy, we performed chronic angiotensin II (AngII) infusion in WT and *Trpm4*^*cKO*^ mice. AngII is a widely used drug to induce cardiac hypertrophy experimentally [27, 38]. We have used mini-osmotic pumps, implanted under the back skin, to deliver 3 mg/kg/day AngII during 2 weeks. As a negative control, we implanted pumps filled with saline in both genotypes. To analyze the hypertrophic effect of AngII, at the end of the experiment, the heart weight:body weight (HW/BW) and heart weight:tibial length (HW/TL) ratios were compared. In the saline-treated groups, no differences were observed between WT and *Trpm4*^*cKO*^ mice. However, in the AngII-treated groups, *Trpm4*^*cKO*^ mice displayed a significant increase in HW/BW and HW/TL ratios, compared to WT mice (Fig. 2a, b). To test age-related spontaneous development of hypertrophy in *Trpm4*^*cKO*^ and WT mice, we measured HW/BW and HW/TL ratios in ~1-year-old mice. We detected no difference in heart weight or in body weight between the two genotypes (Fig. 2c). To test whether the presence of the Cre recombinase in the *Trpm4*^*cKO*^ mice has any effect on the AngII-induced hypertrophic response, we performed AngII infusion experiments on Cre-positive and Cre-negative *Trpm4*^+/+^ mice. As shown in Supplementary Figure 2, the presence of Cre recombinase had no significant effect on HW/BW and HW/TL ratios after AngII treatment in our experimental conditions. Finally, we tested whether the AngII treatment had any effect on TRPM4 expression on the WT heart. To this end, we performed TRPM4-specific qPCR and Western blot (Fig. 2d, e), which showed no difference in the amount of TRPM4 mRNA or protein level between control and AngII-treated WT mice.

### Histological analysis of hypertrophied hearts from WT and *Trpm4*^*cKO*^ mice

**Fig. 3:**
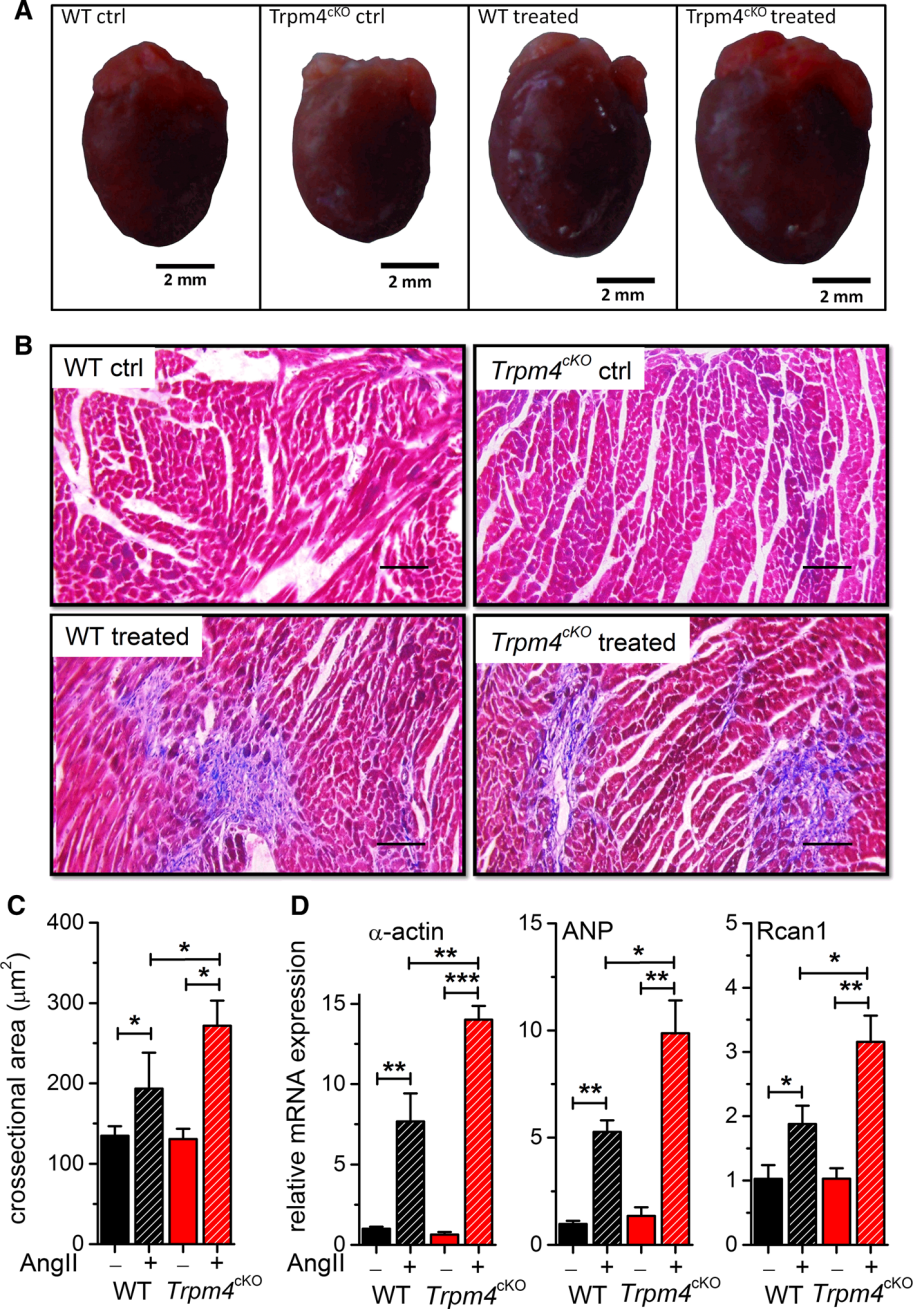
Increased hypertrophic phenotype in *Trpm4* ^*cKO*^ mice compared to WT. **a** Representative pictures from WT and *Trpm4* ^*cKO*^ hearts, after 2 weeks of sham or AngII treatment, as indicated. **b** Representative pictures of Masson’s trichrome-stained left ventricular tissues of WT and *Trpm4* ^*cKO*^ mice with or without AngII treatment. *Blue area* indicates fibrosis. *Scale bar* 50 µm. **c** Cross-sectional area of myocytes after sham or AngII treatment, as indicated. The mean myocyte area was evaluated by measurement of 150 cells per heart (*n* = 3 hearts per group). (mean ± SEM. *p < 0.05). **d** Expression of hypertrophy marker genes (α-actin, ANP and Rcan1) in saline- and AngII-treated groups. Expression was normalized to mTBP and mPGK1 expression. (mean ± SEM; *n* = 8 for each group, *p < 0.05, **p < 0.01)

To further evaluate the hypertrophic response, we performed histological analysis of the cardiac muscle form saline- and AngII-treated mice. Representative pictures from each group are shown in Fig. 3a. The level of cardiac fibrosis was determined with Masson’s trichrome staining. Fibrosis was obvious in the hearts after AngII application (Fig. 3b), but we did not observe differences between the genotypes (Supplementary Figure 3). Instead, the cross-sectional area of WT and *Trpm4*^*cKO*^ myocytes were 193.2 ± 14 and 271.4 ± 12 µm^2^, respectively, after AngII treatment (*n* = 150 cells from 3 mice for each genotype, *p* < 0.05; Fig. 3c).

### Expression of hypertrophy marker genes

To further investigate the altered hypertrophic response in *Trpm4*^*cKO*^ mice, we performed qPCR experiments to evaluate the expression level of known cardiac hypertrophy “marker” genes, i.e., α-actin, myosin heavy chain beta (MYH7), atrial natriuretic peptide (ANP), Rcan1 (a negative regulator of calcineurin of which the transcription is highly dependent on calcineurin–NFAT activation) and the α1H subunit of voltage-gated T-type Ca^2+^ channel (CACNA1H), which is also known to be re-expressed upon cardiac hypertrophy [3]. All these markers showed upregulation after AngII treatment (Supplementary Figure 4), but a specific stronger upregulation of α-actin, ANP and Rcan1 was observed in *Trpm4*^*cKO*^ compared to WT (Fig. 3d).

### TRPM4 deficiency increases the hypertrophic response to AngII of cultured neonatal myocytes

**Fig. 4:**
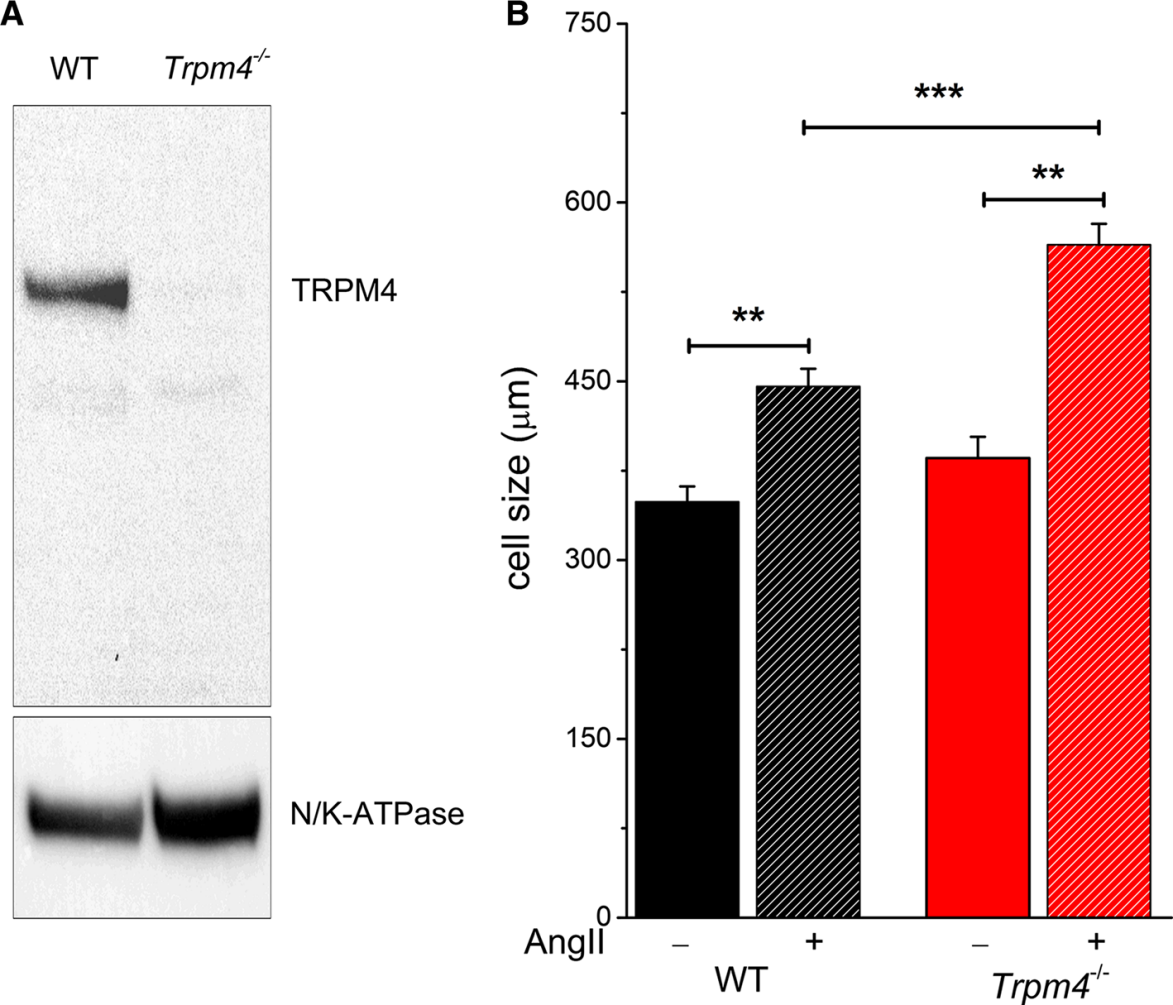
Increased *Trpm4* ^−*/*−^ myocyte hypertrophy in vitro. **a** Representative TRPM4-specific Western blotting from neonatal hearts of WT and *Trpm4* ^−*/*−^ mice. **b** Cell size of WT and *Trpm4* ^−*/*−^ neonatal cardiac myocytes before and after 2 days of AngII (1 µmol/L) treatment. (mean ± SEM; *p < 0.05, **p < 0.01). 50–70 cells were counted from three independent isolations per group

AngII application can have at least two distinct effects on the cardiovascular system, vasoconstriction and subsequent blood pressure elevation, and a direct effect on cardiomyocytes via Ang II receptor type 1 (AT1R). To investigate the direct cellular effect of AngII, we used mouse neonatal ventricular myocytes. Cells were isolated from 1- to 2-day-old pups. The second day after isolation, the culture medium was switched to serum-free medium supplemented with 1 µmol/L AngII. After 2 days incubation with AngII, the cell size was determined. Since neonatal myocytes can have a different gene expression profile compared to adult myocytes, we first tested whether TRPM4 is expressed in WT neonatal cells. Western blotting experiments showed a clear expression of TRPM4 protein in WT and a complete absence in *Trpm4*^−*/*−^ neonatal hearts (Fig. 4a). Similar to our in vivo findings, there was no significant difference between WT and TRPM4^-^deficient cells in surface area under basal condition. However, after 2 days of AngII treatment, the surface area of the WT cells was 445.6 ± 17 µm^2^ compared to 564.2 ± 14 µm^2^ in case of TRPM4^-^deficient cells (50–70 cells from 3 independent preparations for each group, *p* < 0.01) (Fig. 4b).

### Ca^2+^ transients in WT and TRPM4-deficient adult ventricular myocytes

**Fig. 5:**
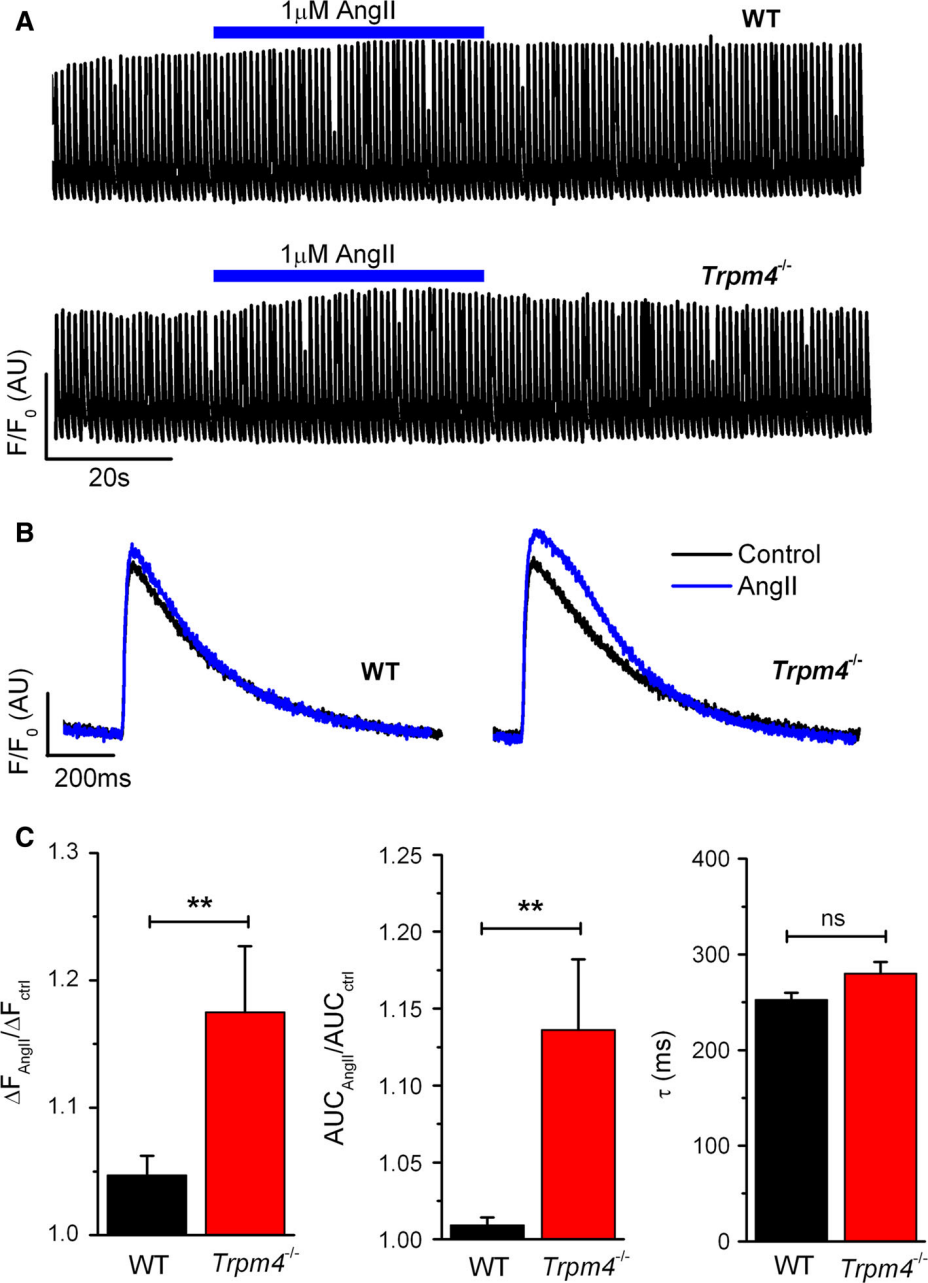
Ca^2+^ transients during AngII application in isolated myocytes**. a** Representative time course of Ca^2+^ transients from paced (1 Hz) WT and *Trpm4* ^−*/*−^ ventricular myocytes. **b** Overlay of representative single traces of Ca^2+^ transients from WT and *Trpm4* ^−*/*−^ myocytes in the presence (*blue*) or absence (*black*) of AngII (1 µmol/L). **c** Comparison of the ratio of the peak value and the AUC of the Ca^2+^ transients under basal and AngII-treated conditions (*left* and *middle* *panel*). Time constant of a mono-exponential fit of the decaying phase of the Ca^2+^ transients during AngII application (*right panel*). (mean ± SEM; *n* = 15–17 from 3 different isolations for each group, *p < 0.05)

To further investigate the cellular effect of AngII, we measured Ca^2+^ transients in intact cardiomyocytes during field stimulation (stimulation frequency 1 Hz). Under basal conditions, Ca^2+^ transients were identical in WT and *Trpm4*^−*/*−^ myocytes. Application of 1 µmol/L AngII to WT myocytes had a very moderate effect on the amplitude and the AUC of the Ca^2+^ transients. However, *Trpm4*^−*/*−^ cells displayed a more pronounced response upon AngII application (Fig. 5a, b). Analyzing the individual Ca^2+^ transients during AngII application, the increase of the peak values in WT and *Trpm4*^−*/*−^ myocytes was 4.8 ± 0.9 and 18.1 ± 5.1 %, respectively (*n* = 15–17 for each group, *p* < 0.05). Similar differences were found when analyzing the area under curve (AUC) of transients: the mean AngII-induced increase was 0.9 ± 0.6 % in WT and 13.6 ± 3.8 % in *Trpm4*^−*/*−^ myocytes (*n* = 15–17 for each group, *p* < 0.05). However, the time constant of the decaying phase was not different between genotypes, suggesting that the Ca^2+^ extrusion mechanisms are unchanged in the *Trpm4*^−*/*−^ myocytes (Fig. 5c).

### Increased store-operated Ca^2+^ entry in *Trpm4*^−*/*−^ adult ventricular myocytes

**Fig. 6:**
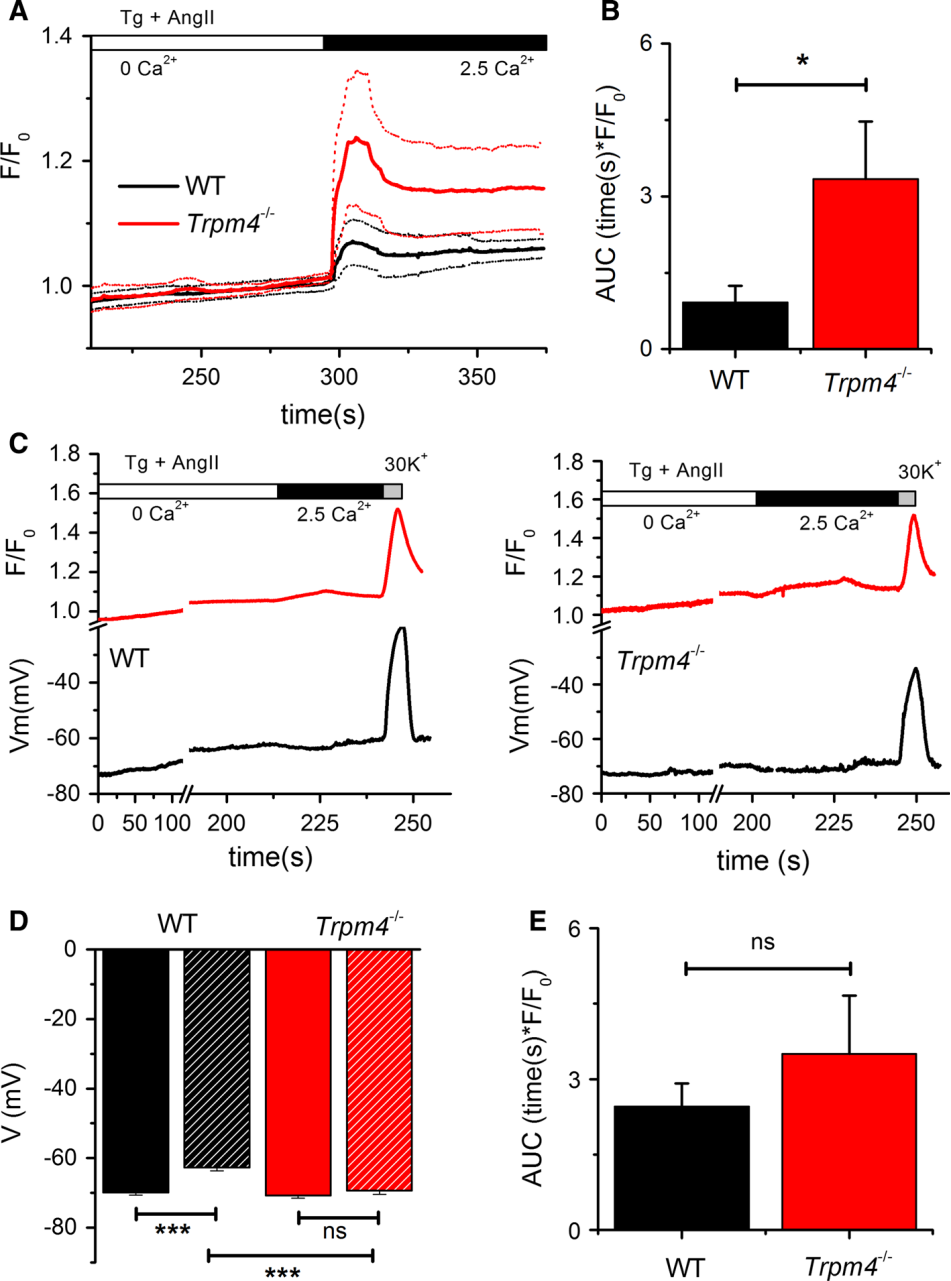
Increased store-operated calcium entry in *Trpm4* ^−*/*−^ myocytes compared to WT. **a** Fluorescence recording of Ca^2+^ entry in WT and *Trpm4* ^−*/*−^ myocytes. The *full* *black* and *red lines* represent the mean fluorescent values from WT and *Trpm4* ^−*/*−^ myocytes, respectively (WT: *n* = 15, *Trpm4* ^−*/*−^: *n* = 16 myocytes were isolated from 3 animals for each group). The dotted lines represent the corresponding SEM values. Solutions were applied as indicated. **b** Comparison of the AUC of fluorescent signal during the Ca^2+^ re-addition phase in panel (**a**). **c** Representative examples of a typical Ca^2+^ re-addition experiment, using extracellular solutions as in panel (**a**). At the end of the experiment 30 mM K^+^ was applied, to test the viability of the cell. Intracellular Ca^2+^ (*red line*) and membrane potential (*black line*) were measured simultaneously. **d** Membrane potential of WT and *Trpm4* ^−*/*−^ myocytes from an experiment as in panel (**c**), at the start of thapsigargin and AngII application (*full colored bar*) and at the maximal Ca^2+^ level after Ca^2+^ re-addition (*patterned bar*) (WT: *n* = 13, *Trpm4* ^−*/*−^: *n* = 14 myocytes were isolated from 4 animals for each group). **e** Comparison of intracellular Ca^2+^ level during Ca^2+^ re-addition, when the membrane potential of the myocytes is clamped at −75 mV (WT: *n* = 8, *Trpm4* ^−*/*−^: *n* = 9) (mean ± SEM in all panels; *p < 0.05; ***p < 0.001, myocytes were isolated from 3 animals for each group)

Recent studies demonstrated that store-operated Ca^2+^ entry (SOCE) plays a key role in mediating cardiomyocyte hypertrophy, both in vitro and in vivo [15, 16, 30, 32, 33]. To test whether the different effect of AngII on the global Ca^2+^ transients can be explained by differences in SOCE between WT and *Trpm4*^−*/*−^ adult ventricular myocytes, we performed a Ca^2+^ re-addition protocol, using AngII to deplete the IP_3_ sensitive stores and thapsigargin to block Ca^2+^ reuptake by SERCA activity. Cells were kept in nominally Ca^2+^-free solution in the presence of 1 µmol/L AngII and 5 µmol/L thapsigargin for 5 min and subsequently perfused with a solution containing 2.5 mmol/L Ca^2+^ to allow SOCE. In these experiments, 30 % of the WT and 65 % of the *Trpm4*^−*/*−^ myocytes displayed an increase in [Ca^2+^]_*i*_ after Ca^2+^ re-addition. The average fluorescent signals from WT and *Trpm4*^−*/*−^ myocytes during Ca^2+^ re-addition is shown in Fig. 6a. Analyzing [Ca^2+^]_*i*_ during the Ca^2+^ re-addition, we found a significantly higher increase in the AUC in *Trpm4*^−*/*−^ myocytes compared to WT (Fig. 6b). This indicates that AngII-mediated SOCE is increased in *Trpm4*^−*/*−^ myocytes.

### Lack of membrane depolarization in *Trpm4*^−*/*−^ adult ventricular myocytes during store depletion

Based on our SOCE experiments, we hypothesized that during AngII application, the release of Ca^2+^ from IP_3_-sensitive stores can activate TRPM4, and the depolarizing current via the channel can reduce the driving force for Ca^2+^ entry via SOCE channels, similar to the case in mast cells [37]. This hypothesis also suggests that TRPM4 activation during the store depletion might lead to moderate depolarization of the resting membrane potential. To test this, we performed membrane potential measurement during the above-discussed Ca^2+^ re-addition protocol, using the perforated patch technique (Fig. 6c). In WT myocytes, we observed a significant 7.3 mV membrane depolarization during the store depletion. In contrast, no significant depolarization was measured in *Trpm4*^−*/*−^ myocytes (Fig. 6d) (WT: *n* = 13; *Trpm4*^−/−^: *n* = 14, *p* < 0.001). In these experiments, we never observed depolarization beyond −60 mV, allowing us to exclude a significant contribution of L-type Ca^2+^ channel activity to the [Ca^2+^]_*i*_ increase during Ca^2+^-reapplication. Furthermore, application of the L-type Ca^2+^ channel blocker nifedipine (1 µmol/L) had no effect on the [Ca^2+^]_*i*_ increase in Ca^2+^ re-addition experiments (Supplementary Figure 5). Application of 30 mmol/L K^+^ at the end of the experiment depolarized the membrane potential and induced a Ca^2+^ transient, indicating that the myocytes are intact and able to maintain their resting membrane potential.

To test whether the difference in [Ca^2+^]_*i*_ increase after Ca^2+^ re-addition between WT and *Trpm4*^−*/*−^ myocytes is mediated by a TRPM4-dependent membrane depolarization, we performed the Ca^2+^ re-addition experiment while clamping the membrane potential at −75 mV. Under this condition, we no longer observed a difference in the rise in [Ca^2+^]_*i*_ between WT and *Trpm4*^−*/*−^ myocytes during the Ca^2+^ re-addition phase (Fig. 6e).

### Increased calcineurin phosphatase activity in hearts of *Trpm4*^*cKO*^

**Fig. 7:**
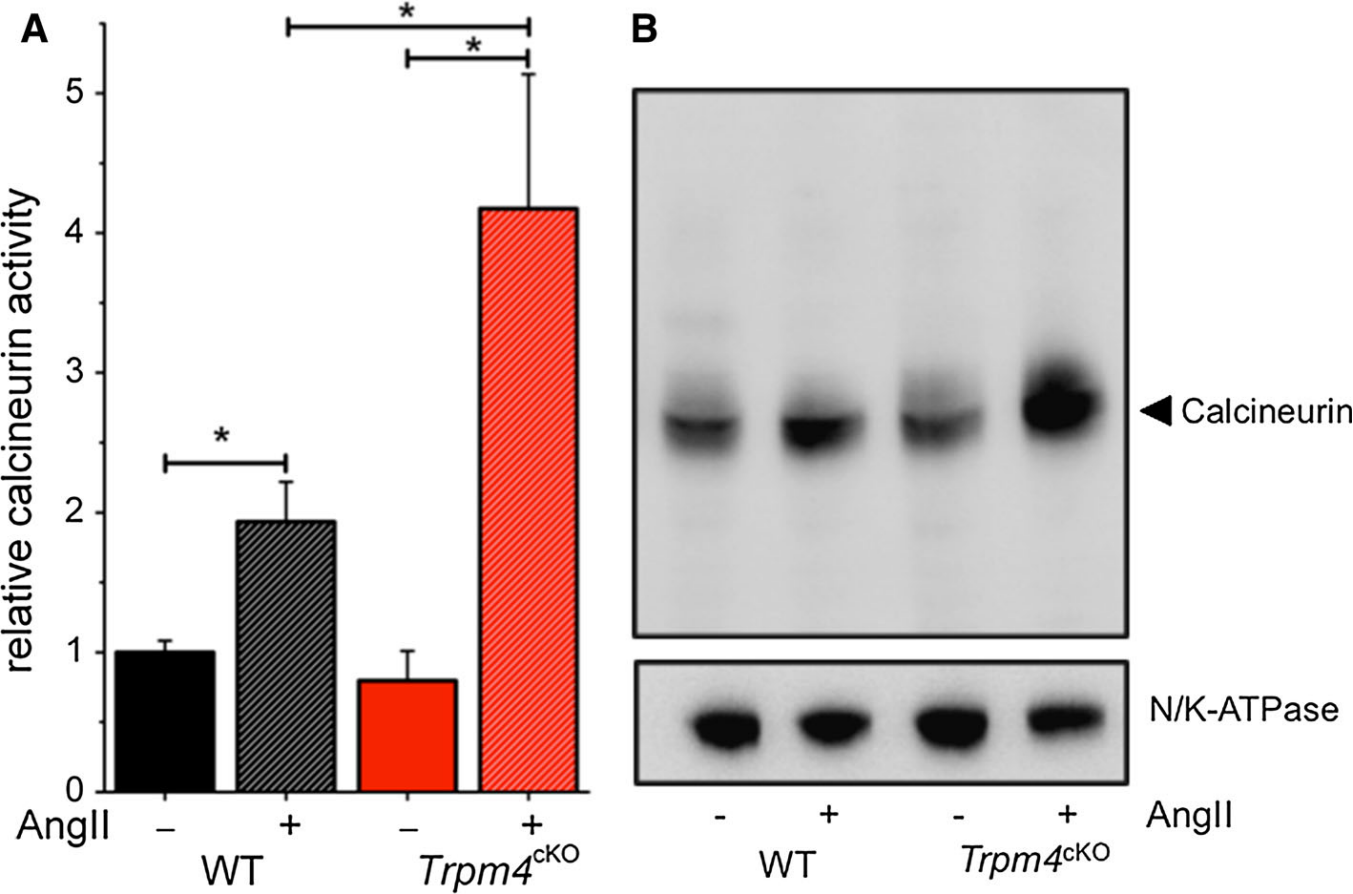
Increased calcineurin enzyme activity in *Trpm4* ^*cKO*^ cardiac muscle compared to WT upon AngII treatment. **a** Calcineurin phosphatase activity, measured from cardiac muscle tissue lysates from WT and *Trpm4* ^cKO^ mice, after sham or AngII treatment, as indicated (mean ± SEM; *n* = 10 for each group, *p < 0.05). **b** Representative calcineurin-specific Western blotting from cardiac muscle lysates of WT and *Trpm4* ^*cKO*^ mice, after sham or AngII treatment, as indicated. Lysates from three hearts per group were pooled in each *lane*

To further investigate the mechanisms behind the altered hypertrophic response in *Trpm4*^*cKO*^ mice, calcineurin enzyme activity was measured. Previously, we have seen increased expression of Rcan1, an endogenous reporter of calcineurin–NFAT activity, in AngII-treated *Trpm4*^*cKO*^ animals (Fig. 3d), which is most likely the result of the enhanced activation of calcineurin [42]. It has been shown previously that the calcium-activated phosphatase calcineurin is both necessary and sufficient to induce cardiac hypertrophy and that both its protein expression and phosphatase activity are increased in response to hypertrophic stimuli [28, 35]. To test calcineurin activity directly, we performed a calcineurin enzyme activity assay. We observed a significant increase in calcineurin activity in the hearts of AngII-treated animals and this increase was even higher in *Trpm4*^*cKO*^ animals (*n* = 10 for each group, *p* < 0.05) (Fig. 7a). However in sham-treated animals, total phosphatase activity was not significantly different between WT and *Trpm4*^*cKO*^ samples (Supplementary Figure 6). To further investigate calcineurin activity, we performed calcineurin-specific Western blot analysis on the same hearts. In line with the increased phosphatase activity, we observed ~1.6 times more protein in WT AngII-treated and ~2.4 times more in *Trpm4*^*cKO*^ AngII-treated animals, whereas there was no significant difference in calcineurin protein level between the genotypes after sham treatment (Fig. 7b). These results suggest that higher SOCE in AngII-treated *Trpm4*^*cKO*^ myocytes leads to an enhanced calcineurin signaling and to a stronger hypertrophic response.

## Discussion

In this study, we demonstrated the effect of the calcium-activated non-selective cation channel TRPM4 on the development of cardiac hypertrophy upon chronic AngII infusion. In our experiments, mice lacking the channel exclusively in the heart were more sensitive to hypertrophic stimulus than WT mice. They displayed a significantly increased heart mass and myocyte cross-sectional area after 2 weeks of chronic AngII treatment. Furthermore, they showed a stronger re-activation of the fetal gene program than WT-treated mice. The increased sensitivity to AngII treatment was also present in vitro in cultured neonatal cardiac myocytes. Finally, we provide evidence that the increased response to AngII treatment was due to the lack of a TRPM4-dependent membrane depolarization leading to increased Ca^2+^ influx via store-operated Ca^2+^ channels. The increased Ca^2+^ influx most likely explains the enhanced activation of the calcineurin–NFAT pathway in *Trpm4*^*cKO*^ myocytes.

AngII is considered as a hypertrophic agent on myocytes since the first description on embryonic chicken cells [1] and later on other species. AngII not only increases blood pressure, through its constrictive effect on the vasculature, but also promotes growth and hypertrophy of cardiac tissue independent of hypertension through the Ang II receptor type 1 (AT1R), which is expressed in cardiomyocytes [27, 38]. Activation of the AT1 receptor triggers a Gq-coupled cascade that results in the formation of IP_3_ and DAG. IP_3_ subsequently stimulates the release of Ca^2+^ from intracellular IP_3_-sensitive Ca^2+^ stores. It has been shown that IP_3_-mediated Ca^2+^ release plays a central role in regulating cardiomyocyte hypertrophy induced by different stimuli, including AngII infusion [29]. On the other hand, apart from Ca^2+^ release, AngII receptor activation will also induce Ca^2+^ influx, via store-operated Ca^2+^ channels, which might involve TRPC channels [10, 33] and/or STIM/ORAI channel complexes [15, 32]. It is increasingly recognized that SOCE is present in cardiomyocytes and serves as an important mechanism in hypertrophy signaling [4]. The presence of SOCE on myocytes has been shown already many years ago [16]. Several publications demonstrated that upon store depletion, SOCE facilitates the influx of Ca^2+^ from the extracellular space to refill intracellular stores, while the resulting increase in intracellular Ca^2+^ concentration also regulates hypertrophy pathways, such as the calcineurin/NFAT cascade [15, 30, 32, 41].

TRPM4 has been described as a regulator of Ca^2+^ influx and Ca^2+^-dependent cell functions in many cell types, including ventricular cardiac myocytes [24, 25, 37]. We have previously shown that TRPM4 proteins are determinants of the inotropic effects of β-adrenergic stimulation by modulating I_CaL_ activity. Moreover, it has been shown that in mast cells TRPM4 is a regulator of Ca^2+^ influx via SOCE channels upon depletion of intracellular Ca^2+^ stores. Since SOCE plays a key role in cardiomyocyte hypertrophy, and TRPM4 is functionally present in mouse ventricular myocytes, we hypothesized that TRPM4 might also regulate the driving force for Ca^2+^ entry during hypertrophic stimulus and therefore could regulate cardiomyocyte hypertrophy signaling.

One major pitfall in research dealing with TRPM4 physiology is the lack of specific inhibitors. 9-Phenantrol has received a lot of attention recently as a TRPM4 antagonist, but it has to be mentioned that the selectivity of this compound on TRPM4 is doubtful. It has been shown recently that 9-phenantrol also activates calcium-activated potassium channels and blocks calcium-activated chloride channels, which limits its usefulness in experiments using primary cells and living animals [2, 11]. To investigate our hypothesis, we generated cardiac-specific *Trpm4*^−/−^ mice (*Trpm4*^cKO^). Notably, *Trpm4*^cKO^ mice do not display hypertension, as was described previously in global *Trpm4*^−/−^ mice [25]. Using the *Trpm4*^cKO^ line, we observed increased heart size after 2 weeks of AngII infusion and also increased myocyte size in histological sections compared to WT. No differences were observed in the saline-treated groups. It was described recently that the global *Trpm4*^−*/*−^ mice display increased HW/BW ratios even without hypertrophic treatment as a result of hyperplasia, but we did not observe this in cardiac-specific *Trpm4*^*cKO*^ mice [6]. Masson trichrome staining showed increased fibrosis after AngII treatment, but this was not different between WT and *Trpm4*^*cKO*^, suggesting that the fibroblast function is not altered by the TRPM4 deletion. We have also found significant increases in the expression levels of several hypertrophy-related genes after AngII treatment in both genotypes, and in case of α-actin, ANP and Rcan1 this increase was significantly higher in *Trpm4*^*cKO*^ mice. ANP and α-actin are commonly up-regulated in case of cardiac hypertrophy; however, Rcan1 is a reporter of calcineurin–NFAT activation, a well described pathway in cardiac hypertrophy. Since the first description in myocytes, this pathway has been shown several times to be necessary and sufficient for the induction of cardiac hypertrophy [28].

Another profound change in *Trpm4*^−*/*−^ myocytes was the altered Ca^2+^ handling during AngII application. As shown before, application of AngII to adult ventricular myocytes had very little effect on calcium transients in case of WT myocytes [18]. However, *Trpm4*^−*/*−^ myocytes responded to AngII application with a significant increase in terms of the amplitude and the AUC of the Ca^2+^ transients. This observation suggests that TRPM4 deletion has an effect on AngII receptor-mediated [Ca^2+^]_*i*_ elevation. In our previous work, we performed a whole-genome mRNA transcript analysis in WT and *Trpm4*^−*/*−^ heart tissue where we found now major up- or downregulation of any genes involved in AngII receptor signaling [24]. However, it has been shown that AngII application can also induce [Ca^2+^]_*i*_ elevation via SOCE channels [30]. Considering that TRPM4 regulates the driving force for SOCE in other cell types, we performed Ca^2+^ re-addition experiments as described earlier [16] to assess directly the magnitude of SOCE in WT and *Trpm4*^−*/*−^ myocytes. We observed increased [Ca^2+^]_*i*_ in *Trpm4*^−*/*−^ myocytes after store depletion by AngII compared to WT cells, which is in line with previous observations in mast cells [37]. As was published before, not every myocyte displays SOCE in this type of assay. The ratio of responding/non-responding myocytes in WT was in line with previously published data. However, the number of responding myocytes was significantly higher in *Trpm4*^−*/*−^ mice and is similar to the response rate of myocytes from hypertrophied hearts [17, 41].

Why do *Trpm4*^−*/*−^ myocytes display increased store-operated Ca^2+^ influx during AngII application? It is well known that TRPM4 activation by high [Ca^2+^]_*i*_ leads to an inward current and the subsequent depolarization can limit the driving force for calcium ions via voltage and non-voltage-gated calcium channels. This has been shown in different cell types including mast cells and cardiac myocytes [24, 37]. Furthermore, Earley and coworkers showed that release of Ca^2+^ from IP_3_-sensitive internal stores can specifically activate TRPM4 and generate a sustained inward current in cerebral smooth muscle cells [12]. Taken together, our data are consistent with the hypothesis that AngII application in cardiac myocytes leads to Ca^2+^ release from IP_3_-sensitive stores, which activates TRPM4, concomitantly with opening of SOCE channels (Supplementary Figure 7). The depolarizing TRPM4 current then limits the driving force for Ca^2+^ entry via the SOCE channels. Consistent with this, when we clamped the membrane potential at −75 mV in myocytes from both genotypes the differences in [Ca^2+^]_*i*_ after Ca^2+^ re-addition was no longer present, suggesting that the original difference was mediated by a TRPM4-dependent membrane depolarization.

Several recent studies showed that Ca^2+^ entry through SOCE channels can activate the calcineurin–NFAT pathway and cause pathological cardiac hypertrophy [16, 30, 32, 41]. Calcineurin is a calcium-dependent phosphatase which, upon activation, dephosphorylates NFAT and promotes its translocation to the nucleus. The activated NFAT initiates the expression of hypertrophy-related genes, including Rcan1. Based on our model, we would expect increased calcineurin–NFAT activation, in line with the observed increased SOCE in *Trpm4*^−*/*−^ myocytes. Indeed, in mRNA expression experiments, we observed increased Rcan1 upregulation in *Trpm4*^*cKO*^ hearts, which is an indicator of the activation of calcineurin–NFAT pathway [42]. To study calcineurin signaling in these hearts, we assessed calcineurin-dependent phosphatase activity and calcineurin expression. It was described previously that the amount of calcineurin protein and subsequently phosphatase activity is enhanced in response to hypertrophic stimuli [35]. Consistent with this, we observed increased enzyme activity in WT hearts treated with AngII, and this increase was more prominent in *Trpm4*^*cKO*^ mice. We observed similar results in terms of the amount of protein, which was increased ~1.6 times in WT and ~2.5 times in *Trpm4*^*cKO*^ animals after AngII infusion. In sham-treated animals, no difference was apparent between both genotypes.

Taken together, our data describe the role of TRPM4 in the development of AngII-induced pathological cardiac hypertrophy. TRPM4, as a Ca^2+^-activated non-selective cation channel negatively modulates the intracellular Ca^2+^ signaling induced by AngII, by limiting the driving force for Ca^2+^ influx via SOCE channels. Therefore, it can fine-tune the amount of calcium activating the calcineurin–NFAT pathway, regulating pathological cardiac hypertrophy.

## Materials and methods

### Mice

To obtain cardiac-specific *Trpm4* knockout mice, *Trpm4*^L3F2^ mice (see Fig. 1) were crossed with EIIa-Cre mice (obtained from the Jackson Laboratory). From the offspring, *Trpm4*^flox^ mice were selected for further breeding. Subsequently, *Trpm4*^flox^ mice were crossed with MLC2a-Cre mice, which resulted in cardiac-specific deletion of exon 15 and 16 of the TRPM4 gene, which encode the first transmembrane domain of the TRPM4 protein. Western blotting confirmed deletion of the protein from the heart (see Fig. 1 in the main text). Mice were routinely genotyped using PCR. For more details on the targeting strategy, see [40]. Experiments were performed on 3- to 6-month-old male C57BL/6 N mice and age- and sex-matched *Trpm4*^−*/*−^ or cardiac-specific *Trpm4* knockout (*Trpm4*^*cKO*^*)* mice. Mice were housed with a 12-h light/12-h dark cycle and allowed water and standard food ad libitum. All animal experiments were performed according to the Guide for the Care and Use of Laboratory Animals (8th edition, National Research Council, USA, 2011) and were approved by the local ethics committee. All mention of *Trpm4*^*cKO*^ in this paper refers to cardiac-specific *Trpm4* knockout mice.

### Adult ventricular cardiomyocytes isolation

8- to 12-week-old mice were heparinized and killed by intraperitoneal injection of pentobarbital (Nembutal, CEVA). The hearts were removed and perfused retrograde for 2 min through the aorta with a Langendorff apparatus at 37 °C with oxygen-saturated nominally calcium-free solution containing (in mmol/L): 117 NaCl, 4 KCl, 1 KH_2_PO_4_, 4 NaHCO_3_, 1.7 MgCl_2_, 10 HEPES, and 10 glucose (pH 7.4). This step was followed by 4–6 min of perfusion with digestion solution [as above, but supplemented with 1 mg/ml collagenase B (Worthington)]. Subsequently, the heart was removed from the setup, atria were discarded and ventricular muscle was minced in collagenase solution (see above). The suspension was filtered through a nylon filter, and cells were centrifuged at 500×*g* for 2 min. The supernatant was discarded and the myocytes were resuspended in the first solution, but supplemented with 1 mmol/L CaCl_2_. Calcium-tolerant, rod-shaped myocytes with clear striations were used for measurements on the day of the isolation.

### Ca^2+^ measurements

For imaging of intracellular Ca^2+^ dynamics, isolated ventricular myocytes were loaded with the Ca^2+^ indicator Fluo-4-AM (1 μmol/L) for 15 min at room temperature. Cells were seeded on glass coverslips in extracellular solution containing (in mmol/L): 118 NaCl, 4.7 KCl, 2.52 CaCl_2_, 1.64 MgSO_4_, 24.88 NaHCO3, 1.18 KH_2_PO_4_, 6 glucose, 2 Na pyruvate. This solution was continuously bubbled with carbogen (5 % CO_2_, 95 % O_2_). Electrical stimulation was achieved using field stimulation. Fluorescence was detected with a photo-multiplier tube and acquired by the Patchmaster software. Excitation light (480 nm) was provided by a Polychrome V monochromator (Till Photonics, Germany).

### SOCE measurement

For store-operated calcium entry measurement, myocytes were handled as above. Myocytes were treated with 1 μmol/L AngII and 5 μmol/L thapsigargin for 5–6 min in nominally Ca^2+^-free buffer to deplete SR stores. Myocytes were then perfused with high Ca^2+^ (2.5 mmol/L), in the continuous presence of thapsigargin and AngII, and store-operated entry was measured as an increase in Fluo-4 fluorescence. For the measurement of the resting membrane potential during SOCE experiment, perforated patch technique was used in current clamp mode. In these experiments the pipette solution contained (in mmol/L) 10 KCl, 10 NaCl, 70 K_2_SO_4_, 1 MgCl_2_ 10 Hepes and 240 µg/ml amphotericin B, pH 7.3.

### Surgical modeling and physiological analysis

8- to 12-week-old mice were anesthetized with isoflurane. Mini-osmotic pumps (Alzet, model: 1002) filled with 3 mg/kg/day Ang II (Calbiochem) or with saline were implanted subcutaneously. Two weeks after the operation, animals were killed by cervical dislocation, hearts were removed, cleaned from fat tissue and big vessels, washed in PBS and weighed. Subsequently, the left tibia was dissected and the length of the bone was measured.

### Isolation of neonatal cardiac myocytes

Neonatal mice (1- to 2-day-old) were used for isolation. Hearts were removed aseptically; atria and big vessels were discarded. The ventricles were cut into small pieces and digested in 0.25 % trypsin for 15 min at 37 °C. Subsequently, supernatants were removed and mixed with an equal volume of HBSS with Ca^2+^ and Mg^2+^. This step was repeated four to five times until the hearts were completely digested. The cells were then cultured in DMEM supplemented with 20 % FBS for 2–3 h, allowing the attachment of non-myocytes. Later, the cells in the supernatant were used and cultured in DMEM supplemented with 20 % FBS. After 1 day, the medium was changed to DMEM without FBS and supplemented with 1 µM AngII for 48 h.

### Calcineurin phosphatase activity assay

Tissue samples from freshly isolated hearts of WT and *Trpm4*^*cKO*^ mice were lysed in calcineurin assay buffer (Enzo Life Science) and centrifuged at 2000×*g*. The supernatant was then snap frozen at −70 °C. The phosphatase activity assay (BML-AK816) was performed based on the manufacturer’s instruction (Enzo Life Science). Calcineurin phosphatase activity was measured spectrophotometrically by detecting free phosphate released from the calcineurin-specific RII phosphopeptide.

### Western blot analysis

Proteins from freshly isolated heart of wild-type and *Trpm4*^*cKO*^ mice were lysed in 1 ml ice-cold lysis buffer (100 mmol/L Tris–HCl, 1 mmol/L MgCl_2_, 0.1 mmol/L phenylmethylsulfonyl fluoride [PMSF] [pH 8] and a protease inhibitors cocktail (Proteoguard™ Clontech) using a Polytron homogenizer. Subsequently, 4 ml saccharose buffer (250 mmol/L saccharose, 10 mmol/L Tris–HCl [pH 7.4], 0.1 mmol/L PMSF and protease inhibitor cocktail) was added to the lysate. The obtained homogenates were centrifuged at 3000×*g* for 15 min to remove any remaining large cellular fragments. The supernatants were ultracentrifuged at 200,000×*g* for 30 min. Pellets containing total membrane fractions were solubilized in a cold saccharose buffer. Protein concentrations were determined by the bicinchoninic acid assay method, using bovine serum albumin (BSA) as a standard. Samples (80 μg) were subjected to SDS-polyacrylamide gel electrophoresis (SDS-PAGE) and subsequently transferred to a polyvinylidene fluoride (PVDF) membrane (Bio-Rad, USA). The respective proteins were detected with purified rabbit polyclonal antibody (Ak578) directed against the amino-terminal end of mouse TRPM4 (*27*), isoform-specific mouse monoclonal calcineurin-Aß (sc365612; Santa Cruz Biotechnology) and monoclonal mouse anti-Na^+^/K^+^ ATPase (1: 5000 dilution) (Abcam, UK) antibodies. Immunoreactive complexes were visualized by chemiluminescence, using anti-rabbit IgG (Sigma, USA) or anti-mouse IgG (GE Healthcare) antibodies conjugated to horseradish peroxidase.

### Histology

Four mice of each genotype were perfused with saline. Then the hearts were fixed with zinc-based fixative solution (BD Pharmingen, USA). Subsequently, hearts were processed and embedded in paraffin wax. Serial sections were cut to a thickness of 5–7 μm and documented using a Zeiss microscope (Axiovert 40). For Masson’s trichrome staining, a commercially available kit (Sigma-Aldrich) was used based on the supplier’s instructions. For the analysis of the obtained pictures, ImageJ (NIH, USA) software was used.

### Analysis of gene expression

After euthanasia by cervical dislocation, hearts were removed, washed in ice-cold PBS, immediately snap frozen in liquid nitrogen and kept at −80 °C until final processing. Total RNA was extracted using the RNeasy Mini Kit (Qiagen), following the manufacturer’s protocol. RNA concentration and quality were assessed using the Experion RNA StdSens Analysis Kit (Bio-Rad). For cDNA synthesis, Ready-To-Go You-Prime First-Strand Beads (GE Healthcare) were used based on the supplier’s protocol. Generated cDNAs were stored at −20 °C. qPCR reactions, composed of cDNA template, Universal TaqMan MasterMix (2× concentrated, Life Technologies), TaqMan assay (20× concentrated, Life Technologies) and H_2_O, were performed with the 7500 Fast Real-Time PCR System (Life Technologies). mPGK1 and mTBP, selected using the geNorm application, were used as endogenous controls. TaqMan assays used: CACNA1H-Mm00445382_m1, TRPM4-Mm00613173_m1, ACTA1-Mm00808218_g1, ANP-Mm01255748_g1, RCAN1-Mm01213407_m1, MYH7-Mm00600555_m1.

### Statistics

All statistical analyses were performed using Prism (Graphpad) or Origin (Microcal, USA) software. Data are expressed as mean ± SEM, unless otherwise specified. Differences between groups were compared by two-sample *t* test and two-way repeated measurement ANOVA followed by a Tukey post hoc test, unless mentioned otherwise. When assumptions were not valid, non-parametric tests were performed (Mann–Whitney test, Friedman ANOVA). A *p* value <0.05 was considered to be statistically significant.

## Electronic supplementary material

Supplementary material 1 (PDF 1084 kb)

## References

[1] Baker KM, Aceto JF (1990). Angiotensin II stimulation of protein synthesis and cell growth in chick heart cells. Am J Physiol.

[2] Burris SK, Wang Q, Bulley S, Neeb ZP, Jaggar JH (2015). 9-phenanthrol inhibits recombinant and arterial myocyte TMEM16A channels. Br J Pharmacol.

[3] Chiang CS, Huang CH, Chieng H, Chang YT, Chang D, Chen JJ, Chen YC, Chen YH, Shin HS, Campbell KP, Chen CC (2009). The Ca(v)3.2 T-type Ca(2+) channel is required for pressure overload-induced cardiac hypertrophy in mice. Circ Res.

[4] Collins HE, Zhu-Mauldin X, Marchase RB, Chatham JC (2013). STIM1/Orai1-mediated SOCE: current perspectives and potential roles in cardiac function and pathology. Am J Physiol Heart Circ Physiol.

[5] Demion M, Bois P, Launay P, Guinamard R (2007). TRPM4, a Ca^2+^-activated nonselective cation channel in mouse sino-atrial node cells. Cardiovasc Res.

[6] Demion M, Thireau J, Gueffier M, Finan A, Khoueiry Z, Cassan C, Serafini N, Aimond F, Granier M, Pasquie JL, Launay P, Richard S (2014). Trpm4 gene invalidation leads to cardiac hypertrophy and electrophysiological alterations. PLoS One.

[7] Fonfria E, Murdock PR, Cusdin FS, Benham CD, Kelsell RE, McNulty S (2006). Tissue distribution profiles of the human TRPM cation channel family. J Recept Signal Transduct Res.

[8] Frey N, Katus HA, Olson EN, Hill JA (2004). Hypertrophy of the heart: a new therapeutic target?. Circulation.

[9] Frey N, Olson EN (2003). Cardiac hypertrophy: the good, the bad, and the ugly. Annu Rev Physiol.

[10] Gao H, Wang F, Wang W, Makarewich CA, Zhang H, Kubo H, Berretta RM, Barr LA, Molkentin JD, Houser SR (2012). Ca(2+) influx through L-type Ca(2+) channels and transient receptor potential channels activates pathological hypertrophy signaling. J Mol Cell Cardiol.

[11] Garland CJ, Smirnov SV, Bagher P, Lim CS, Huang CY, Mitchell R, Stanley C, Pinkney A, Dora KA (2014). TRPM4 inhibitor 9-phenanthrol activates endothelial cell intermediate conductance calcium-activated potassium channels in rat isolated mesenteric artery. Br J Pharmacol.

[12] Gonzales AL, Amberg GC, Earley S (2010). Ca^2+^ release from the sarcoplasmic reticulum is required for sustained TRPM4 activity in cerebral artery smooth muscle cells. Am J Physiol Cell Physiol.

[13] Guinamard R, Bois P (2007). Involvement of transient receptor potential proteins in cardiac hypertrophy. Biochim Biophys Acta.

[14] Heineke J, Molkentin JD (2006). Regulation of cardiac hypertrophy by intracellular signalling pathways. Nat Rev Mol Cell Biol.

[15] Hulot JS, Fauconnier J, Ramanujam D, Chaanine A, Aubart F, Sassi Y, Merkle S, Cazorla O, Ouille A, Dupuis M, Hadri L, Jeong D, Muhlstedt S, Schmitt J, Braun A, Benard L, Saliba Y, Laggerbauer B, Nieswandt B, Lacampagne A, Hajjar RJ, Lompre AM, Engelhardt S (2011). Critical role for stromal interaction molecule 1 in cardiac hypertrophy. Circulation.

[16] Hunton DL, Lucchesi PA, Pang Y, Cheng X, Dell’Italia LJ, Marchase RB (2002). Capacitative calcium entry contributes to nuclear factor of activated T-cells nuclear translocation and hypertrophy in cardiomyocytes. J Biol Chem.

[17] Hunton DL, Zou L, Pang Y, Marchase RB (2004). Adult rat cardiomyocytes exhibit capacitative calcium entry. Am J Physiol Heart Circ Physiol.

[18] Ikenouchi H, Barry WH, Bridge JH, Weinberg EO, Apstein CS, Lorell BH (1994). Effects of angiotensin II on intracellular Ca^2+^ and pH in isolated beating rabbit hearts and myocytes loaded with the indicator indo-1. J Physiol.

[19] Inoue R, Jensen LJ, Shi J, Morita H, Nishida M, Honda A, Ito Y (2006). Transient receptor potential channels in cardiovascular function and disease. Circ Res.

[20] Jacobs G, Oosterlinck W, Dresselaers T, Geenens R, Kerselaers S, Himmelreich U, Herijgers P, Vennekens R (2015). Enhanced beta-adrenergic cardiac reserve in Trpm4^−^/^−^ mice with ischaemic heart failure. Cardiovasc Res.

[21] Kruse M, Schulze-Bahr E, Corfield V, Beckmann A, Stallmeyer B, Kurtbay G, Ohmert I, Brink P, Pongs O (2009). Impaired endocytosis of the ion channel TRPM4 is associated with human progressive familial heart block type I. J Clin Invest.

[22] Launay P, Fleig A, Perraud AL, Scharenberg AM, Penner R, Kinet JP (2002). TRPM4 is a Ca^2+^-activated nonselective cation channel mediating cell membrane depolarization. Cell.

[23] Makarewich CA, Correll RN, Gao H, Zhang H, Yang B, Berretta RM, Rizzo V, Molkentin JD, Houser SR (2012). A caveolae-targeted L-type Ca^2+^ channel antagonist inhibits hypertrophic signaling without reducing cardiac contractility. Circ Res.

[24] Mathar I, Kecskes M, Van der Mieren G, Jacobs G, Camacho Londono JE, Uhl S, Flockerzi V, Voets T, Freichel M, Nilius B, Herijgers P, Vennekens R (2014). Increased beta-adrenergic inotropy in ventricular myocardium from Trpm4^−/−^ mice. Circ Res.

[25] Mathar I, Vennekens R, Meissner M, Kees F, Van der Mieren G, Camacho Londono JE, Uhl S, Voets T, Hummel B, van den Bergh A, Herijgers P, Nilius B, Flockerzi V, Schweda F, Freichel M (2010). Increased catecholamine secretion contributes to hypertension in TRPM4-deficient mice. J Clin Invest.

[26] Mills RW, Milan DJ (2010). TRPM4-linked isolated cardiac conduction defects: bad trafficking causes electrical gridlock. Circ Cardiovasc Genet.

[27] Miyata S, Haneda T (1994). Hypertrophic growth of cultured neonatal rat heart cells mediated by type 1 angiotensin II receptor. Am J Physiol.

[28] Molkentin JD, Lu JR, Antos CL, Markham B, Richardson J, Robbins J, Grant SR, Olson EN (1998). A calcineurin-dependent transcriptional pathway for cardiac hypertrophy. Cell.

[29] Nakayama H, Bodi I, Maillet M, DeSantiago J, Domeier TL, Mikoshiba K, Lorenz JN, Blatter LA, Bers DM, Molkentin JD (2010). The IP3 receptor regulates cardiac hypertrophy in response to select stimuli. Circ Res.

[30] Nakayama H, Wilkin BJ, Bodi I, Molkentin JD (2006). Calcineurin-dependent cardiomyopathy is activated by TRPC in the adult mouse heart. FASEB J.

[31] Nilius B, Vennekens R (2006). From cardiac cation channels to the molecular dissection of the transient receptor potential channel TRPM4. Pflugers Arch.

[32] Ohba T, Watanabe H, Murakami M, Sato T, Ono K, Ito H (2009). Essential role of STIM1 in the development of cardiomyocyte hypertrophy. Biochem Biophys Res Commun.

[33] Onohara N, Nishida M, Inoue R, Kobayashi H, Sumimoto H, Sato Y, Mori Y, Nagao T, Kurose H (2006). TRPC3 and TRPC6 are essential for angiotensin II-induced cardiac hypertrophy. EMBO J.

[34] Stallmeyer B, Zumhagen S, Denjoy I, Duthoit G, Hebert JL, Ferrer X, Maugenre S, Schmitz W, Kirchhefer U, Schulze-Bahr E, Guicheney P (2012). Mutational spectrum in the Ca(2+)—activated cation channel gene TRPM4 in patients with cardiac conductance disturbances. Hum Mutat.

[35] Taigen T, De Windt LJ, Lim HW, Molkentin JD (2000). Targeted inhibition of calcineurin prevents agonist-induced cardiomyocyte hypertrophy. Proc Natl Acad Sci USA.

[36] Uhl S, Mathar I, Vennekens R, Freichel M (2014). Adenylyl cyclase-mediated effects contribute to increased Isoprenaline-induced cardiac contractility in TRPM4-deficient mice. J Mol Cell Cardiol.

[37] Vennekens R, Olausson J, Meissner M, Bloch W, Mathar I, Philipp SE, Schmitz F, Weissgerber P, Nilius B, Flockerzi V, Freichel M (2007). Increased IgE-dependent mast cell activation and anaphylactic responses in mice lacking the calcium-activated nonselective cation channel TRPM4. Nat Immunol.

[38] Wada H, Zile MR, Ivester CT, Cooper G, McDermott PJ (1996). Comparative effects of contraction and angiotensin II on growth of adult feline cardiocytes in primary culture. Am J Physiol.

[39] Watanabe H, Murakami M, Ohba T, Takahashi Y, Ito H (2008). TRP channel and cardiovascular disease. Pharmacol Ther.

[40] Wettschureck N, Rutten H, Zywietz A, Gehring D, Wilkie TM, Chen J, Chien KR, Offermanns S (2001). Absence of pressure overload induced myocardial hypertrophy after conditional inactivation of Galphaq/Galpha11 in cardiomyocytes. Nat Med.

[41] Wu X, Eder P, Chang B, Molkentin JD (2010). TRPC channels are necessary mediators of pathologic cardiac hypertrophy. Proc Natl Acad Sci USA.

[42] Yang J, Rothermel B, Vega RB, Frey N, McKinsey TA, Olson EN, Bassel-Duby R, Williams RS (2000). Independent signals control expression of the calcineurin inhibitory proteins MCIP1 and MCIP2 in striated muscles. Circ Res.

