# Supplementary material for: The Ca2+-activated cation channel TRPM4 is a negative regulator of angiotensin II-induced cardiac hypertrophy
Source: Basic Res Cardiol. 2015 Jun 5;110(4):43. doi: 10.1007/s00395-015-0501-x (PMC4456993; doi:10.1007/s00395-015-0501-x)
Supplement: Supplementary file 1 — Supplementary material 1 (PDF 1084 kb) [file 395_2015_501_MOESM1_ESM.pdf]

Supplemental Material

Basic Research in Cardiology

The Ca^{2+} activated cation channel TRPM4 is a negative regulator of Angiotensin II induced cardiac hypertrophy

Miklós Kecskés, Griet Jacobs, Sara Kerselaers, Ninda Syam, Aurélie Menigoz, Peter Vangheluwe, Marc Freichel, Veit Flockerzi, Thomas Voets and Rudi Vennekens[*]

* To whom correspondence should be addressed:

Rudi Vennekens

Laboratory of Ion Channel Research, Department of Molecular and Cellular Medicine, KU Leuven, Campus Gasthuisberg, Herestraat 49, B-3000 Leuven, Belgium

tel: ++32-16-330 218,

fax:++32-16-330 732

email: rudi.vennekens@med.kuleuven.be

Supplementary Figure 1

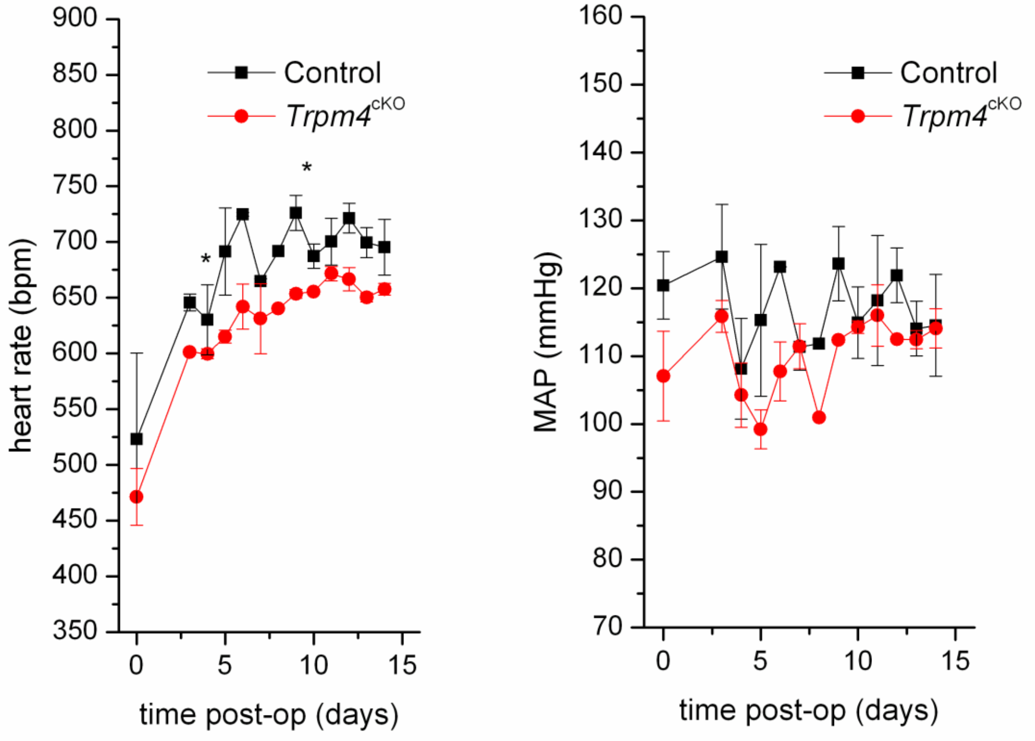

Hemodynamic parameters are not affected by TRPM4 deletion from heart tissue. Heart rate **(A)** and mean arterial pressure **(B)** are identical between *Trpm4*^{cKO} and control littermates over the 2 weeks period after transmitter implantation. Mean ± SEM.; n=3 for each group.

Supplementary Figure 2

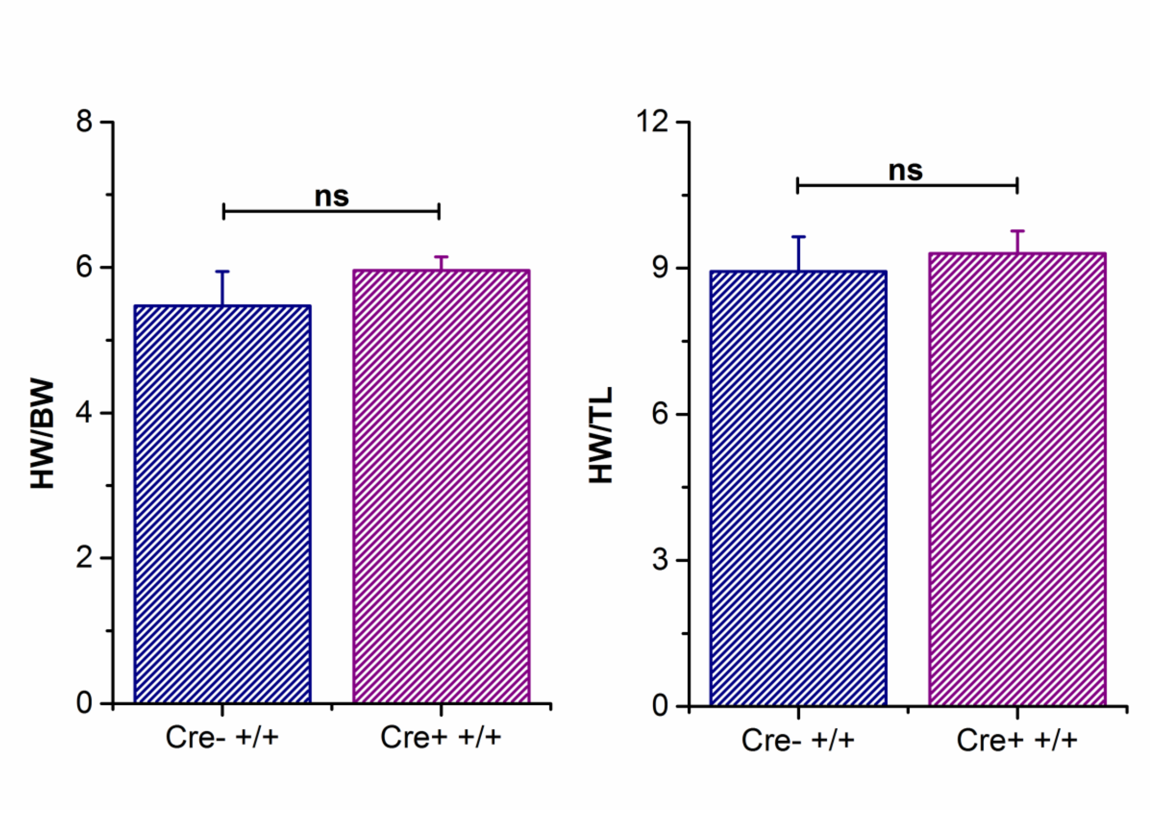

Cre-recombinase has no effect on AngII induced hypertrophic response. Heart weight/body weight (HW/BW) and heart weight/tibial length (HW/TL) ratios of $Cre^{-}/^{+/+}$ and $Cre^{+}/^{+/+}$ mice after Ang II (3 mg/kg/day during 2 weeks via osmotic pumps) treatment. At the time of the experiment (8-12 weeks of age) body weight was not different between groups. (mean ± SEM; $Cre^{-}/^{+/+}$: n=3 $Cre^{+}/^{+/+}$: n=4).

Supplementary Figure 3

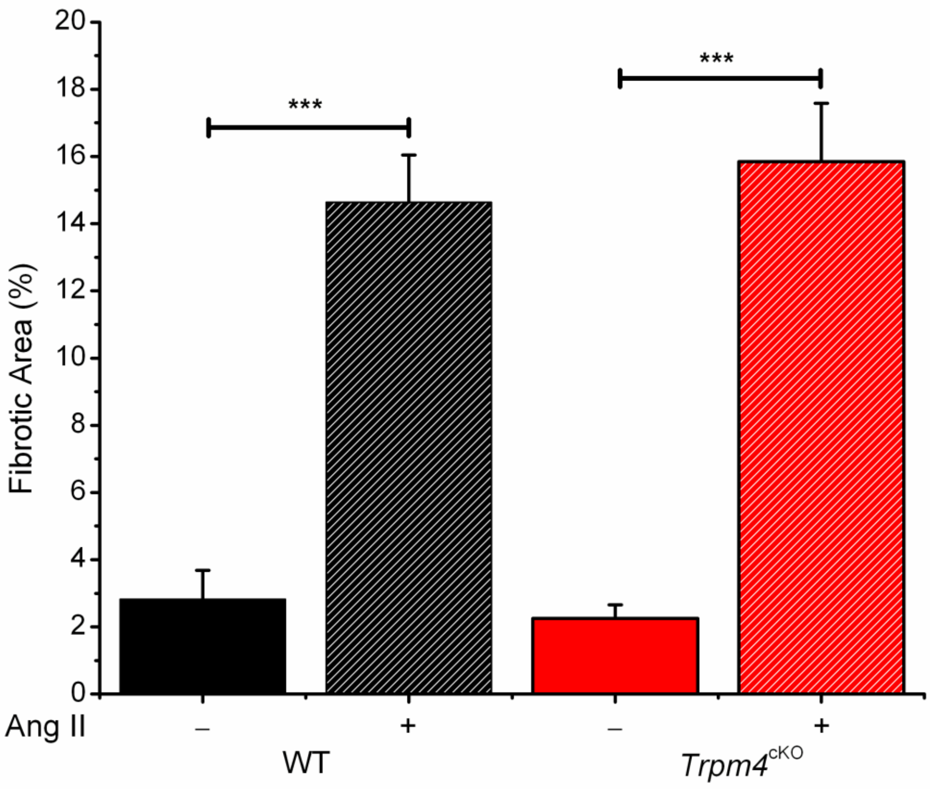

Fibrotic area is not different between WT and *Trpm4*cKO hearts after AngII treatment. Percentage of blue area on Masson's Trichrome stained left ventricular tissues with or without AngII treatment. Fibrotic area was determined by measurement of blue area on slices from hearts as indicated (n = 5 hearts per group) using ImageJ (NIH) software. Mean ± SEM., ***: $p<0.001$.

Supplementary Figure 4

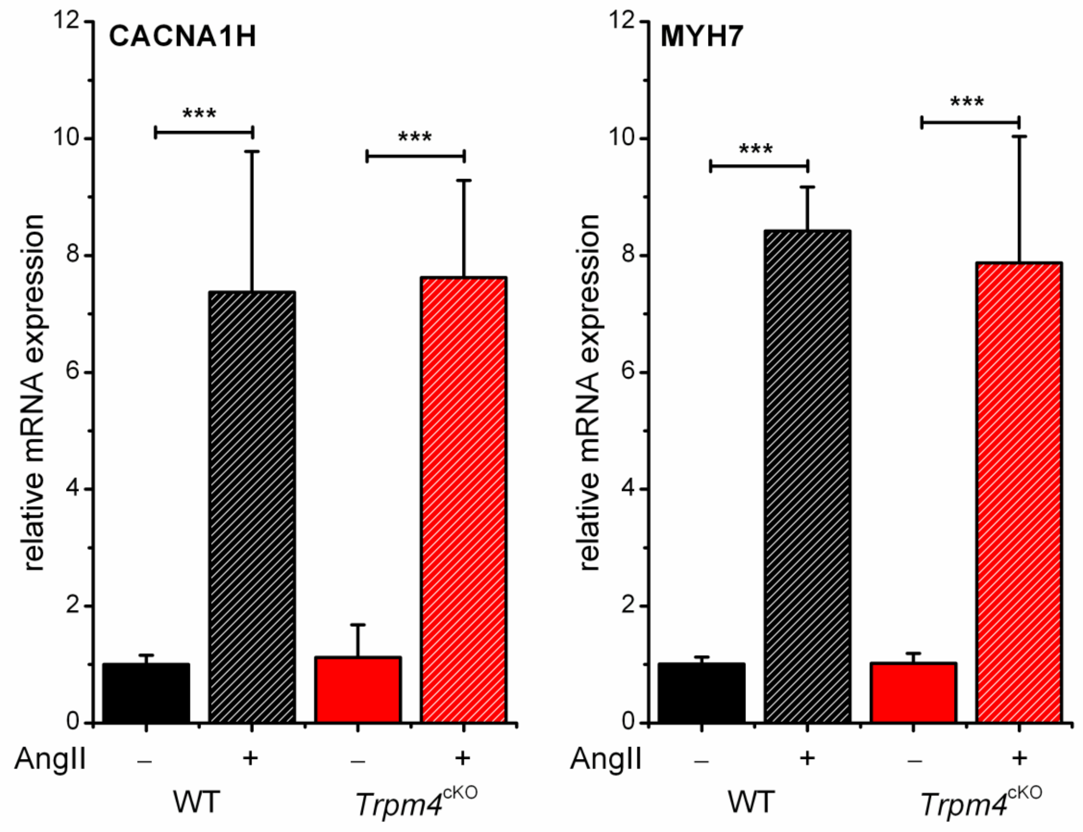

Increased expression of hypertrophy marker genes after AngII treatment. Expression of CACNA1H and MYH7 genes are increased after AngII treatment but no differences were observed between WT and *Trpm4*^{cKO} mice. Expression was normalized to mTBP and mPGK1 expression. Mean ± SEM.; n=8 for each group, *: p<0.05.

Supplementary Figure 5

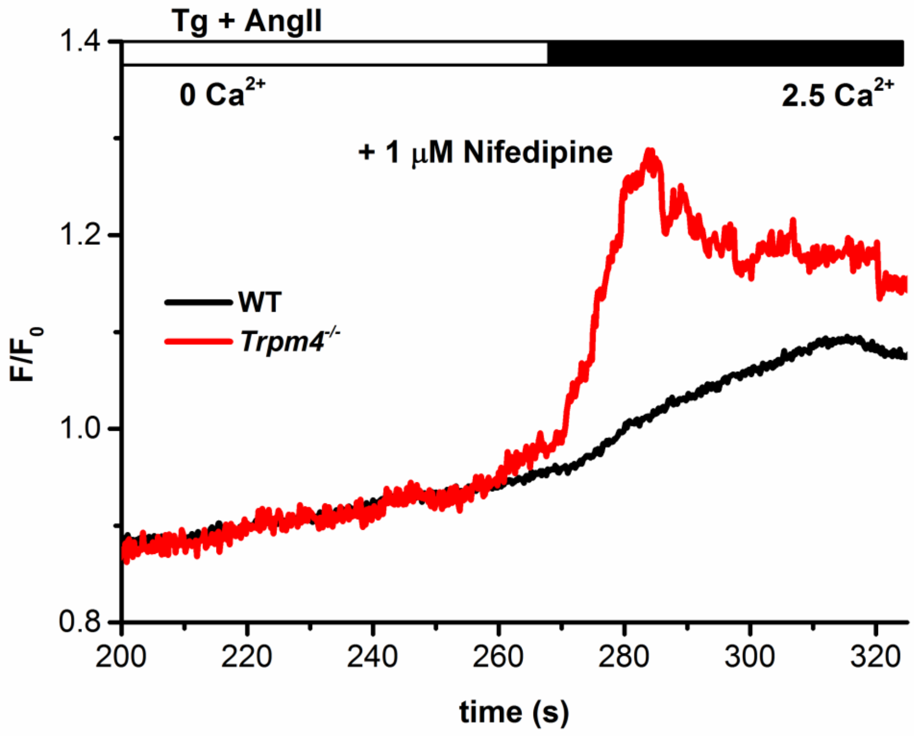

Nifedipine has no effect on store operated calcium entry. Representative fluorescence recordings of Ca^{2+} entry in WT and *Trpm4*$^{-/-}$ myocytes in the continuous presence of 1 µM Nifedipine (WT: n = 15, *Trpm4*$^{-/-}$: n = 10, ~14% of WT and ~50% of *Trpm4*$^{-/-}$ myocytes displayed an increase in $[Ca^{2+}]_i$ after Ca^{2+} re-addition).

Supplementary Figure 6

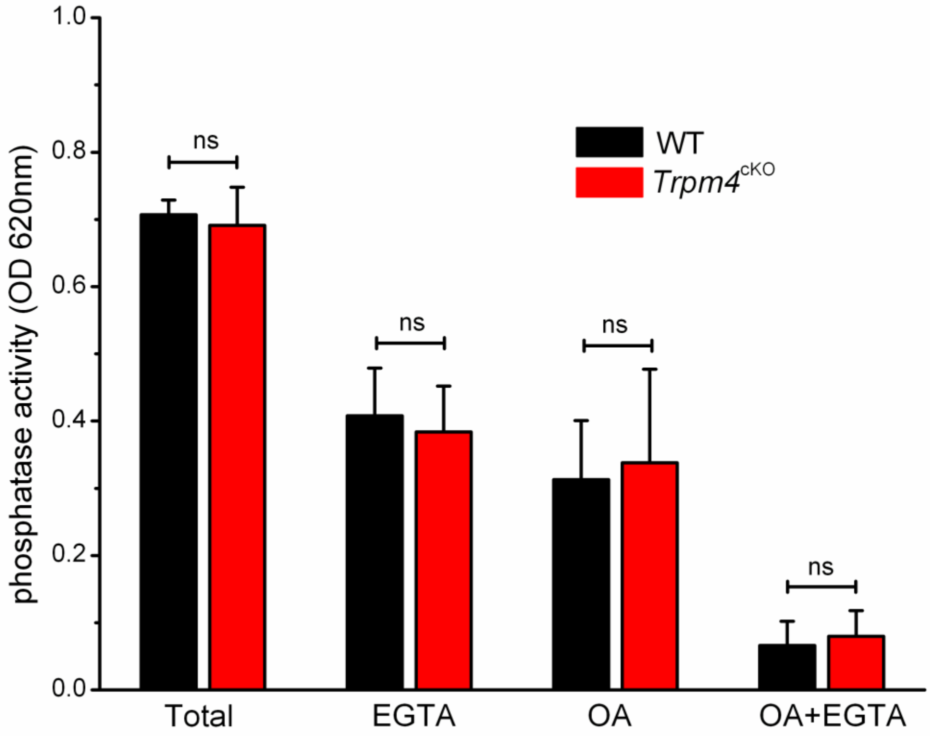

Baseline phosphatase activity is unaltered in *Trpm4*^{cKO} heart tissue. Comparison of phosphatase activity measured from lysate of heart tissue as indicated. First group indicates total activity, second indicates activity in the presence of EGTA (i.e. calcium independent phosphatase activity), third indicates activity in the presence of Okadaic Acid (i.e. phosphatase activity excluding PP1 and PP2A phosphatases) and fourth indicates activity in the presence of EGTA+OA. Mean ± SEM.; n=3 for each group.

Supplementary Figure 7

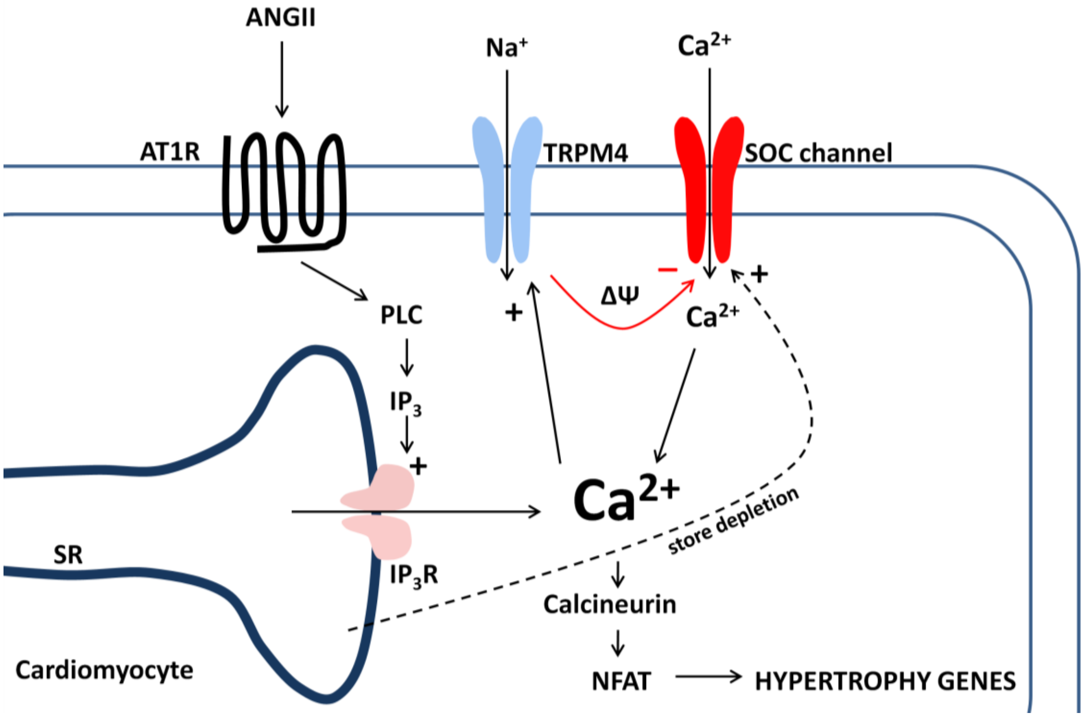

Proposed model illustrating the role of TRPM4 in cardiac hypertrophy. Stimulation of the Gq coupled AT1 receptor activates PLC and increases the cytosolic Ca^{2+} concentration by releasing Ca^{2+} from IP_3-sensitive stores. The released Ca^{2+} activates TRPM4; on the other hand the store depletion will activate SOCE channels. The activated TRPM4 will depolarize the membrane potential which will negatively affect the driving force for Ca^{2+} entry via SOC channels. Deletion of TRPM4 eliminates this negative effect and the increased Ca^{2+} entry via SOCE channels will lead to increased calcineurin-NFAT signaling.
